# Identification, Comparison, and Profiling of Selected Diarrhoeagenic Pathogens from Diverse Water Sources and Human and Animal Faeces Using Whole-Genome Sequencing

**DOI:** 10.3390/microorganisms13061373

**Published:** 2025-06-12

**Authors:** Arinao Murei, Maggy Ndombo Benteke Momba

**Affiliations:** Department of Environmental, Water and Earth Sciences, Arcadia Campus, Tshwane University of Technology, 175 Nelson Mandela Avenue, Arcadia, Pretoria 0001, South Africa

**Keywords:** whole-genome sequencing (WGS), conventional PCR, MALDI-TOF MS, water sources, rural communities

## Abstract

Consumption of contaminated drinking water is known to cause waterborne diseases such as diarrhoea, dysentery, typhoid, and hepatitis. This study applied whole-genome sequencing (WGS) to detect, identify, compare, and profile diarrhoeagenic pathogens (*Vibrio cholerae*, Shiga toxin-producing *Escherichia coli*, and *Escherichia coli* O157:H7) from 3168 water samples and 135 faecal samples (human and animal). Culture-based methods, MALDI-TOF mass spectrometry, and PCR were employed prior to WGS for identification of pathogens. Culture-based results revealed high presumptive prevalence of STEC (40.2%), *V. cholerae* (37.1%), and *E. coli* O157:H7 (22.7%). The MALDI-TOF confirmed 555 isolates with *V. cholerae* identified as *Vibrio albensis*. Shiga toxin-producing *Escherichia coli* (STEC) was more prevalent in wastewater (60%), treated water (54.1%), and groundwater (36.8%). PCR detected 46.4% of virulence genes from the water isolates and 66% of virulence genes from the STEC stool isolates. WGS also revealed STEC (92.9%) as the most prevalent species and found common virulence (e.g., *hcp1/tssD1* and *hlyE*) and resistance (e.g., *acrA* and *baeR*) genes in all three types of samples. Five resistance and thirteen virulence genes overlapped among treated water and stool isolates. These findings highlight the diarrhoeagenic pathogens’ public health risk in water sources and underscore the need for better water quality monitoring and treatment standards.

## 1. Introduction

Tracking and monitoring the spread of diarrhoeagenic pathogens is crucial for effective public health interventions, especially in rural communities where access to healthcare resources may be limited. Diarrhoeal diseases continue to pose a substantial burden on global health, contributing to high morbidity and mortality rates, particularly among vulnerable populations [1]. Waterborne pathogens pose significant risks to public health worldwide, causing a range of diseases and outbreaks [2]. Identifying and characterising these pathogens is crucial for effective surveillance, outbreak response, and the implementation of appropriate control measures. Rural communities, in particular, face unique challenges when it comes to the surveillance and management of diarrhoeal diseases [3]. Limited access to healthcare facilities, inadequate sanitation infrastructure, and poor water quality contribute to increased susceptibility to diarrhoeagenic pathogens.

Consumption of contaminated drinking water can cause waterborne diseases such as diarrhoea, dysentery, typhoid, and hepatitis A [4]. These waterborne diseases are caused by bacteria such as *Vibrio cholerae*, *Shigella*, and *Salmonella* spp. and protozoan parasites such as *Giardia* spp. and *Cryptosporidium* spp., among others. For this study, two bacteria were selected, namely *Vibrio cholerae* and *Escherichia coli*. Several studies have identified and detected the virulome and resistome of these bacteria using different methods, including WGS [5,6,7,8,9]. For *E. coli*, this study focused on Shiga toxin-producing *E. coli* (STEC), including *E. coli* O157:H7, which has been reported to cause human diseases and outbreaks and has been found in healthy cattle, sheep, and goats [10,11,12,13]. By elucidating the genomic characteristics and transmission patterns of these pathogens, we can inform public health strategies and interventions aimed at reducing the burden of diarrhoeal diseases in vulnerable communities and clinical guidance on suitable treatment regimens.

Traditional methods for pathogen detection and profiling often rely on culture-based techniques and targeted testing, which have limitations in terms of sensitivity, specificity, and the ability to capture the genetic diversity of pathogens [14,15]. In recent years, the advent of next-generation sequencing technologies, particularly whole-genome sequencing (WGS), has revolutionised the field of pathogen identification and characterisation. In addition, WGS allows for the comprehensive analysis of the entire genetic material of microorganisms, providing high-resolution insights into their genome composition, serotype, antimicrobial resistance, and virulence factors [12]. By leveraging the power of WGS, researchers can gain a deeper understanding and comprehensively analyse diarrhoeagenic pathogens circulating in rural communities.

In this study, we applied the culture-dependent method for these two selected bacterial pathogens (*V. cholerae* and *E. coli*) to diverse water sources, although a significant number of studies have generally described the conventional method as time-consuming and laborious. Thereafter, PCR and WGS were used to provide a broad knowledge of the microorganisms studied and offered some advantages, such as the detection of multiple genes simultaneously and cost-effectivity. Moreover, molecular methods meet the requirements for the reliable analysis of pathogenic bacteria. These include high specificity, high sensitivity, good reproducibility, and automation.

The objective of this study was to advance our understanding of the genome characteristics and the distribution of waterborne pathogens among various water sources used by communities in rural areas. Through the identification and comparison of pathogen genomes, we can gain insights into the transmission dynamics, variations in virulence factor genes (VF genes), and antimicrobial resistance (AMR) profiles of these organisms. Such information is vital for improving water quality management, implementing targeted interventions, and preventing waterborne disease outbreaks. Overall, the use of whole-genome sequencing (WGS), the most widely used form of next-generation sequencing (NGS), for waterborne pathogen surveillance represents a powerful tool for public health and environmental monitoring. By enhancing our ability to identify, compare, and profile pathogens from diverse water sources, this study contributes to the development of more effective strategies for preventing and controlling waterborne diseases, ultimately safeguarding public health and promoting water safety.

## 2. Materials and Methods

### 2.1. Ethical Consideration and Study Areas

This study was approved by the Tshwane University of Technology (TUT) through its Faculty Committee for Research Ethics (FCRE 2019/09/011 (FCPS 03) (SCI)). The purpose of the investigation was explained to the Vhembe District Municipality in order to access all water sources and permission was granted by the municipal committee. Permission was also obtained from the tribal authorities or chief in selected villages, and consent was obtained from the selected households at the beginning of the project.

### 2.2. Sample Collection

Surface water, groundwater, treated drinking water, wastewater and stool samples were collected from different sources in three local municipalities, namely the Thulamela Local Municipality, Collins Chabane Local Municipality, and Makhado Local Municipality of the Vhembe District Municipality. A random sampling approach was employed to select sampling points in terms of water source type and location. The matrices included surface water (R and D), groundwater (S, B, and DUG), treated water (TWPC), stored water (HC), wastewater (SP), and stool samples (SS) of humans and animals (SS) from areas surrounding water sources and from pit latrines to track and compare the pathogens detected in faeces with those found in water samples (Table S1). After collection, all samples were stored in an insulated cooler box with ice packs and transported to the Microbiology Laboratory at Tshwane University of Technology (TUT), where analyses were performed within 24 h.

### 2.3. Enrichment and Isolation of Diarrhoeagenic Pathogens

To isolate Shiga toxin-producing *Escherichia coli* (STEC), Oxoid™ Tryptone Soya Broth (Thermo Fisher Scientific in Johannesburg, South Africa) was prepared according to the manufacturer’s instructions. Prior to water sample collection, dilutions were made using autoclaved distilled water; the dilution volumes are shown in Table S2. Approximately 1 mL of water samples and wastewater effluent was added to 9 mL of the prepared Tryptone Soya Broth (TSB). Human and animal stool samples (approximately 3 g) were mixed with 9 mL of brain heart infusion broth for growth enrichment (Thermo Fisher Scientific in Johannesburg, South Africa). The mixture was then incubated at 37 °C for 18–24 h. Subsequently, 100 µL of the incubated broth was streaked onto plates containing CHROMagar™ O157 for *E. coli* O157:H7 isolation and CHROMagar™ STEC for non-O157 STEC isolation, both obtained from MediaMage, Johannesburg, South Africa. The plates were incubated at 37 °C for 18–24 h. Distinctive pink to mauve colonies on the plates represented *E. coli* O157:H7 and non-O157 STEC. For *Vibrio cholerae* isolation, 100 mL of a water sample was filtered using a 0.45 µm membrane filter (Merck, Darmstadt, Germany). The filters, along with 1 mL of wastewater and 1 mL of prepared stool solution, were separately placed in test tubes containing double-strength alkaline peptone water (pH 8.5) (Merck, Darmstadt, Germany and Merck, Johannesburg, South Africa). The test tube was vigorously shaken and incubated for 6–8 h at 37 °C. Afterwards, 100 µL of the overnight culture was streaked onto plates containing CHROMagar™ *Vibrio* supplemented with 0.95% alkaline peptone water (APW). The plates were incubated aerobically for 24 h at 37 °C. Blue colonies observed on the media indicated the presence of *V. cholerae*. Positive controls for STEC (ATCC 43888) and *V. cholerae* (ATCC 14035) were obtained from the TUT Microbiology Laboratory. All colonies (pink to mauve colonies for STEC and *E. coli* O157:H7 and blue colonies for *V. cholerae*) were isolated and purified for further molecular analysis.

### 2.4. Bacterial Identification

#### 2.4.1. Mass Spectrometry Technology (MALDI-TOF)

The identification of bacterial isolates involved the use of matrix-assisted laser desorption ionisation time-of-flight (MALDI-TOF) mass spectrometry technology [16]. This analysis was performed at the MALDI-TOF diagnostic service located at the Department of Microbiology and Plant Pathology, University of Pretoria, South Africa, using the MALDI-TOF Mass Spectrometer (Bruker Daltonics GmbH & Co., Bremen, Germany). This method was performed following the procedure described by Ramaite et al. [17]. Positive isolates identified through MALDI-TOF MS were selected for further molecular analysis.

#### 2.4.2. Conventional PCR for Identification of Target Pathogens

For molecular study, it is important to mention that only one bacterial isolate per positive samples was taken into consideration for a specific target bacteria.

a. DNA extraction

Of the 555 (17%) presumptive positive samples determined by culturing, *n* = 223 (40.2%) were STEC, *n* = 126 (227%) were *E. coli* O157:H7, and *n* = 206 (37.1%) were *V. cholerae*. The MALDI-TOF MS results confirmed that most of these isolates were STEC [24% (*n* = 36.8)], followed by *E. coli* O157:H7 [15.7% (*n* = 87)] and *V. cholerae* [13.7% (*n* = 76). The preserved bacterial isolates were thawed and subsequently centrifuged at 13,000× *g* for 1 min. The ZymoBIOMICS™ DNA Miniprep Kit from Zymo Research (Inqaba Biotechnical Industries, Pretoria, South Africa) was utilised to extract the total genomic DNA from the bacterial pellets, following the instructions provided by the manufacturer. To determine the quantity and quality of the extracted DNA, a NanoDrop^TM^ 2000 spectrophotometer (Thermo Scientific in Johannesburg, South Africa) was employed. The genomic DNA (gDNA) suspension obtained was stored at −80 °C until further analysis, either through polymerase chain reaction (PCR) or whole-genome sequencing (WGS).

b. DNA Amplification by conventional multiplex PCR

To detect *E. coli* and *V. cholerae*, all bacterial isolates in this study underwent further analysis using conventional multiplex polymerase chain reaction (PCR) amplification. Each multiplex PCR (mPCR) reaction was conducted in a total volume of 25 μL. This volume included the following reagents: 5 μL of template DNA, 0.5 μL of forward primer (10 M), 0.5 μL of reverse primer (10 M), 12.5 μL of NEB Taq 2X master mix, and 6.5 μL of nuclease-free water, which were obtained from Inqaba Biotechnical Industries, Pretoria, South Africa. The amplification process was carried out using a MiniAmp™ Plus Thermal Cycler (Thermo Fisher Scientific, Johannesburg, South Africa). For the detection of Shiga toxin-producing *Escherichia coli* (STEC), a multiplex PCR assay targeting four (4) important virulence-associated genes, namely *stx1*, *stx2*, *eae*, and *hlyA*, was performed. The identification of *E. coli* O157:H7 involved the detection of three (3) virulence-associated genes: *rfb_O157_*, *fliCH7*, and *eaeA. Vibrio cholerae* was detected using three (3) virulence-associated genes: *ctxAB*, *tcpA*, and *ompW.* Detailed descriptions of the primers and annealing temperatures are given in Table 1. Following PCR amplification, the products were separated using electrophoresis on a 1% agarose gel prepared in 1× TAE buffer, as described by Ramaite et al. [17].

#### 2.4.3. Library Preparation and Sequencing

Library preparation and sequencing of single isolate procedures were carried out at the Agricultural Research Council Biotechnology Platform in Pretoria, South Africa, utilising the Illumina HiSeq^®^ 2500 platform. Initially, the quantity and quality of the DNA were assessed using the Qubit™ dsDNA BR assay kit from Invitrogen, Thermo Fisher Scientific, Waltham, MA, USA. Approximately 2 µg of isolated genomic DNA from each single isolate was employed for constructing Illumina paired-end libraries using the Illumina Nextera XT DNA library preparation method. The generated libraries underwent validation through quality control checks to ensure their suitability for sequencing. Barcodes were assigned to all qualified libraries, and sequencing was performed in accordance with the manufacturer’s instructions. This study employed whole-genome sequencing (WGS) rather than metagenomic sequencing to obtain high-resolution genetic profiles for single bacterial isolates. The aim of the sequencing process was to achieve an average genome coverage of 30× or greater for all isolates. The resulting sequence data were obtained in FASTQ format files and subsequently subjected to bioinformatic analysis.

#### 2.4.4. Bioinformatic Analysis

To assess the quality of the raw sequence reads, FastQC v.0.11.9 [21] was utilised for quality control, including the removal of low-quality sequences. Trimmomatic (v0.36) [22] was employed to trim the reads by removing adapter sequences and low-quality regions. The UCHIME algorithm [23] was applied to eliminate any human DNA contamination. Initially, the Kraken 2 v2.1.0 software program was used to identify the most abundant bacteria in the raw reads. Subsequently, the assembled scaffolds underwent analysis with autoMLST to determine the most closely related species. A species was considered identifiable if the average nucleotide identity (ANI) was equal to or greater than 0.96. In cases where the ANI fell below 0.96, the closest species was indicated as either *E. coli* or *V. cholerae*. The raw BLAST^®^ outputs were further filtered based on three (3) criteria to improve result reliability: (i) a minimum length of 100 base pairs (length = qEnd to qStart); (ii) a percentage pairwise sequence identity (PIDE) greater than 70; and (iii) a query coverage exceeding 70. De novo assembly of the raw reads was performed using SPAdes De Novo Assembler Software v.1.03 [24]. The quality of the assembly was assessed using QUAST version 5.3.0 [25]. The assembled sequences were submitted to the NCBI Sequence Read Archive with BioProject accession number PRJNA964706. Gene recognition was conducted using Prodigal v.2.6.3 [26]. Functional annotation was conducted to identify antimicrobial resistance (AMR) determinants and virulence factors (VFs) by querying the CARD database v.1.2.1 and VFDB database [27], respectively. An AMR and VF determinant was considered part of the core resistome if it was present in all the assessed matrices.

### 2.5. Statistical Analysis

The prevalence disparity among the matrices surface water (R and D), groundwater (S, B, and DUG), treated water (TWPC), stored water (HC), wastewater (SP), and stool samples (SS) was evaluated using the chi-square (χ^2^) test. To determine the relative abundance of antimicrobial resistance genes (ARGs) and virulence factors (VFs) or virulence factor (VF) genes, the results were presented as heat maps. Graphical visualisations were created using Excel (2019) and the Venny (version 2.1) tool online.

## 3. Results

### 3.1. Prevalence of Potential Diarrhoeagenic Pathogens by Culture-Based Methods

A total of 3303 samples were collected from various sources comprising surface water, groundwater, treated water, stored water, wastewater, and stool samples. The detailed sample collection data for dry and wet seasons are given in Table S1. The culture-based results indicate varying levels of potential STEC, *E. coli* O157:H7, and *V. cholerae* contamination in various water sources and stool samples (Table 2). Surface water had comparatively high contamination with 23.5% and 22.8% detection of potential STEC and *V. cholerae*, respectively. Groundwater and treated water had low rates of contamination with potential STEC in 3.8% and 2.7% of samples, respectively, and potential *V. cholerae* was isolated from 1.4% and 2.3%, demonstrating relatively good microbial quality. Stored water, on the other hand, depicted increased contamination with potential STEC at 5.9% and *V. cholerae* at 5.4%, probably resulting from recontamination following treatment. Potential *E. coli* O157:H7 was found to be more prevalent in wastewater, at 12.5% (*n* = 13), among all water samples, with the highest prevalence of 30.4% in stool samples. Potential STEC and *V. cholerae* were isolated in 8.7% and 17.3% wastewater samples, confirming them as critical pathogen transmission sources. Stool samples also captured the highest prevalence, with potential STEC detected in 32.6% and *V. cholerae* in 33.3%, confirming human infection and potential disease transmission. Overall, the results put surface water, wastewater, and stored water into the spotlight as major contamination hotspots, while treated and groundwater sources recorded comparatively lower microbial dangers.

### 3.2. Isolates Confirmed as Diarrhoeagenic Pathogens

Of the 555 presumptive positive samples determined by culturing methods, *n* = 349 (62.9%) for *E. coli* and (*n* = 206 (37.1%) for *V. cholerae*, the MALDI-TOF MS Biotyper^®^ results confirmed that 223 (40.2%) isolates were STEC, 126 (27%) isolates were *E. coli* O157:H7, and 206 (37.1%) isolates of *V. cholerae* were identified as *V. albensis.* The MALDI-TOF results also revealed high variations in the identification of targeted pathogens in various water sources and stool samples (Table 3). The highest presence of STEC was observed in wastewater (60%), followed by treated water (54.1%) and groundwater (36.8%). Stored water showed a moderate contamination rate of 42.6%, which is higher than groundwater, pointing to recontamination during storage. *Escherichia coli* O157:H7 showed the overall lowest prevalence, ranging from 0.7–2.8% in water samples and at 1.1% in stool samples. *Vibrio cholerae* occurred in lower rates across all sample types, with the highest in treated water (21.6%) and groundwater (14.7%). Wastewater and stool samples had 17.5% and 16.2% *V. cholerae* detection rates, respectively, suggesting human exposure and faecal shedding of the pathogen. This study also revealed that among the isolates categorised as presumptive *E. coli*, several other bacterial species, such as *Plesiomonas shigelloides* (1.4%; *n* = 5) and *Proteus mirabilis* (0.9%; *n* = 3), were identified and the remaining 14.3% (*n* = 50) could not be identified. For presumptive *V. cholerae*, the remaining isolates were mostly *Aeromonas* spp. (18.45%; *n* = 38), *Pseudomonas aeruginosa* (9.71%; *n* = 20), *Pseudomonas mendocina* (8.74%; *n* = 18), *Morganella morganii* (7.3%; *n* = 15), and *Enterobacter cloacae* (5.8%; *n* = 12) and the remaining 13.1% (*n* = 27) could not be identified.

### 3.3. Abundance of Virulence-Associated Genes Identified from Various Matrices by PCR

Overall, among the isolates that were confirmed as *E. coli* by MALDI-TOF MS, the PCR results revealed that 6.4% (*n* = 147) and 26.2% (*n* = 83) of isolates were from water samples and 66% (*n* = 33) and 24% (*n* = 12) were from stool samples, which harboured one or more virulence-associated genes specific to STEC and *E. coli* O157:H7, respectively. Additionally, 14.8% (*n* = 47) of isolates from water samples and 36% (*n* = 18) of isolates from stool samples possessed one or more virulence-associated genes of *V. cholerae*. Overall, STEC was detected in 49% of all samples, with the highest prevalence in stool samples (66%) followed by surface water (50%), wastewater (50%), and stored water samples (48.1%). For *E. coli* O157H:7, 26.2% (*n* = 83) originated from water samples and 24.0% (*n* = 12) from stool samples. *Vibrio cholerae* was found in 17.7% of total samples, with the highest prevalence in stool samples (36.0%), implying active *V. cholerae* infection. Surface water (22.2%) and wastewater (17.5%) also showed significant contamination. Detection in treated water was 17.1% and in groundwater was 12.1%. None of the spring water samples tested positive for *V. cholerae* (Table S3 in Supplementary Data). Notably, a total of 34 isolates (9.1%) did not harbour any of the target genes (*stx1*, *stx2*, *eae*, *hlyA*, *rfbO157*, *fliCH7*, *eaeA*, *ctxA*, *tcpA*, and *ompW*) for either STEC, *E. coli* O157:H7, or *V. cholerae*.

**Figure 1:**
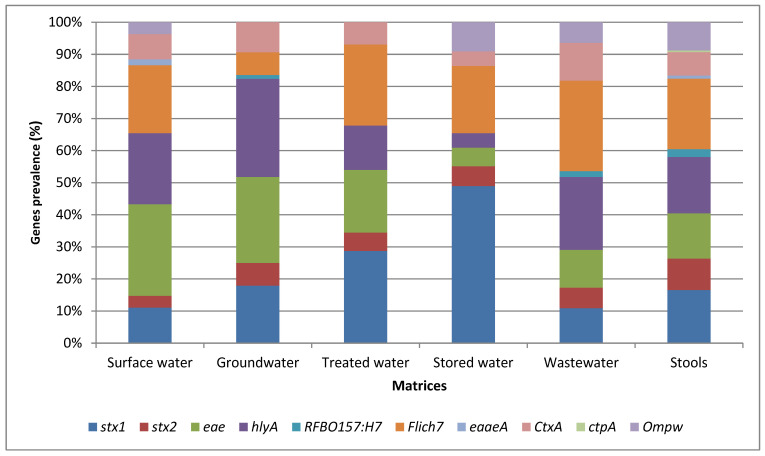
Relative abundance of the most abundant virulence-associated genes identified in the various matrices assessed by conventional PCR.

Figure 1 illustrates the relative abundance of the virulence-associated genes identified in the various matrices assessed by conventional PCR. Among all the target genes, the *stx1* gene detected in STEC exhibited the highest prevalence of 72.1% from stored water. The *stx2* gene was found to have the highest prevalence of 40% in STEC isolated from stool samples. The highest prevalence of *fliCH_7_* isolated in *E. coli* O157:H7 was detected from stool samples [*n* = 45 (90%)], while the highest prevalence of *hlyA* in STEC isolated in water samples was detected at 62.5% from groundwater samples. Furthermore, the *eae* gene exhibited the highest prevalence of 77.5% in STEC isolates from surface water samples. The least frequently detected virulence-associated genes in the isolates from the various matrices were *tcpA* and *eaeA* (carried by *V. cholerae*) and *rfb_O157_* (carried by *E. coli* O157:H7) (Figure 1).

### 3.4. Bioinformatic Analysis

Conventional PCR results revealed that isolates from each source were positive and were showing similar patterns of genes. These isolates were regarded as containing similar organisms; consequently, their DNA was pooled to make one sample. A total of 35 pooled samples were subjected to whole-genome sequencing (WGS) using the HiSeq 2500 platform. Of these, there were 74.3% (*n* = 26) of STEC, 5.7% (*n* = 2) of *E. coli* O157:H7, and 20% (*n* = 7) of *V. cholerae*. *Vibrio cholerae* isolates yielded very low reads due to insufficient sequencing depth. For *E. coli*, the raw sequence data obtained from water samples and stool samples were collected and pre-processed to remove any noise, ensuring the quality and the reliability of the subsequent analysis. Sequencing resulted in the generation of over 7.13 gigabytes (Gb) of unzipped data for functional annotations. The raw reads used in this study have been deposited in the NCBI (accession number PRJNA1123913). On average, each sample yielded approximately 1,635,181 raw reads. The summary statistics provided include sample ID, number of contigs, total length of each base pair (bp), number of sequences, N50, guanine–cytosine (GC) content (%), CDS, and GenBank accession number (provided in Table 4. The resulting genome assemblies of *E. coli* ranged between 1,255,779 and 3,968,282 bp in size. Hand-dug wells had the highest number of contigs (approximately 3588 contigs); the lowest number of contigs was observed in septic tank wastewater (1429 contigs). The N50 values for all assemblies ranged from 705 bp to 1224 bp. All 28 samples of *E. coli* genomes sequenced in the present study contained an average GC content of 52%. The genome coverage for all samples was >30×. Table 5 gives a summary of the genome assemblies of *E. coli*.

#### 3.4.1. Identification

The AutoMLST results revealed that the most abundant bacterial genera were *E. coli* [100% (*n* = 28)], where 92.9% (*n* = 26) were STEC isolates and 7.1% (*n* = 2) were *E. coli* O157:H7 isolates. However, we were not able to distinguish between STEC and *E. coli* O157:H7 due to lower coverage (<30×), which had led to incomplete O/H antigen gene detection. For *V. cholerae*, only one (1) out of seven (7) sequences was closely related to *V. cholerae*, with an ANI = 0.96. Of the 26 STEC sequences, most of them were from the groundwater (*n* = 8) and stored water (*n* = 6), followed by stool samples (*n* = 4), wastewater (*n* = 4), surface water (*n* = 3), and treated water (*n* = 1). The two sequences for *E. coli* O157:H7 were each from surface water and a stool sample.

#### 3.4.2. Most Abundant Virulence Factors (VFs)

**Figure 2:**
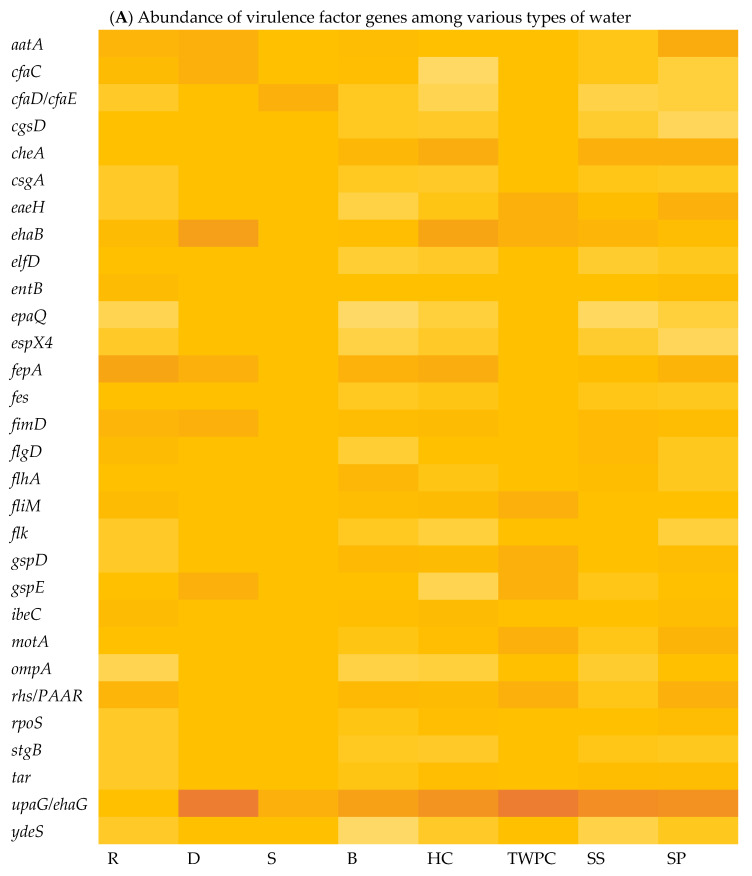

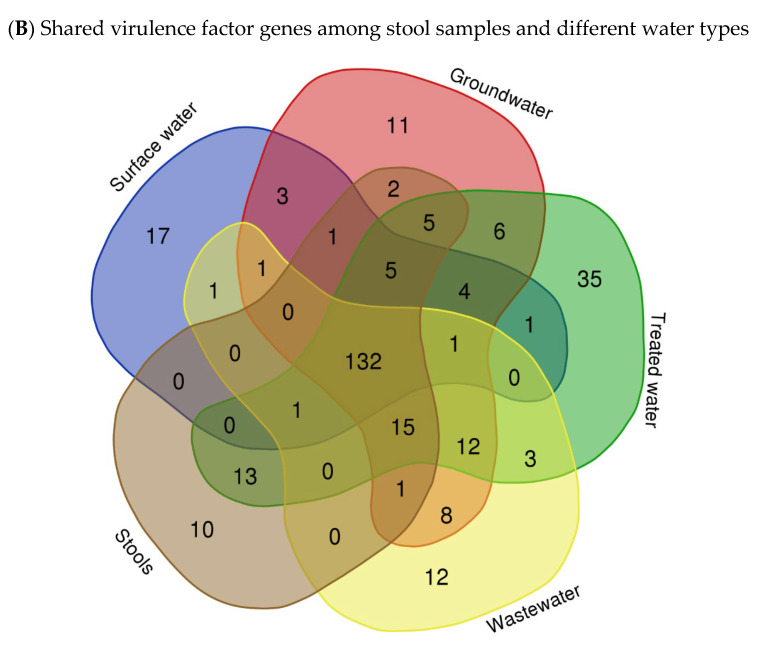
Heat map showing the abundance of VF genes of *E. coli* isolated in various types of water (**A**) (with colour gradient ranges from pale orange (low or zero abundance) to intermediate shades (moderate abundance) to dark/deep orange (high abundance)) and Venn diagram showing the number of shared VF genes among stool samples and different types of water (**B**).

The virulome of *E. coli* isolates was analysed, indicating variations in the virulence factor genes (VF genes). A total of 153 VF genes across all the samples were identified using virulome analysis. Among the different sample types, the septic tank wastewater (SP) exhibited the highest number of VF genes, with a count of 118, followed by stool samples (SS) with 116 VF genes and the household water storage containers (HC) with 115 VF genes, while the spring water (S) samples exhibited the lowest number of VF genes at 18. In Figure 2A, which illustrates the relative abundance of VF genes in the *E. coli* isolates in the various types of water, it can be observed that *upaG* and *ehaG* (shown as *upaG/ehaG* on the heat map) were consistently the most frequently identified VF genes in the majority of the *E. coli* isolates, occurring 92 times, followed by the *ehaA*, *fepA*, and *cheA* VF genes, which were detected with counts of 52, 50, and 47, respectively. In contrast, *gspM* was the least identified VF gene, detected in only four (4) *E. coli* isolates. Furthermore, the *hcp1/tssD1*, *hlyE*, *mrkA*, and *mrkB* genes encoding virulence factors were each detected five (5) times in very few *E. coli* isolates. The most frequent VF genes identified, like *upaG/ehaG*, are associated with adhesion mechanisms, while *ehaA* is implicated in autotransporter-mediated pathogenesis and *fepA* encodes a siderophore receptor implicated in iron acquisition [12]. Several VF genes known to contribute to the virulence and pathogenicity of *E. coli* leading to diarrhoeal diseases were detected, including c*faC*, *cfaD/cfaE*, *csgA*, *eaeH*, *espX4*, *fepA*, *fimD*, *ibeC*, and *ompA* genes. Moreover, the findings indicated that a total of 132 VF genes were shared between the stool samples (SSs) and all the different types of water sources analysed, including surface water (R and D), treated (drinking) water (TWPC and HC), groundwater (S and B), and wastewater (SP) (Figure 2B). Stool samples shared 13 VF genes with the drinking water in households, but only two (2) VF genes with groundwater. Wastewater and groundwater shared eight (8) VF genes. Fifteen VF genes were simultaneously identified in stools, wastewater, and drinking water in households. Around 12 VF genes were detected in wastewater, groundwater, and drinking water in households. Groundwater and drinking water shared six (6) VF genes. However, no shared VF genes were detected among *E. coli* in surface water, groundwater, stools, and wastewater.

#### 3.4.3. Most Abundant Antibiotic Resistance Genes (ARGs)

**Figure 3:**
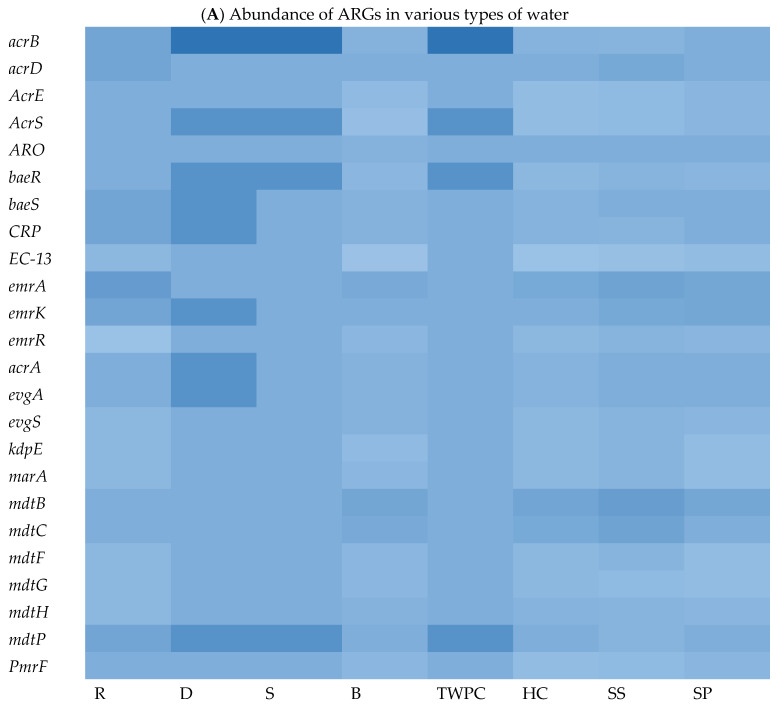

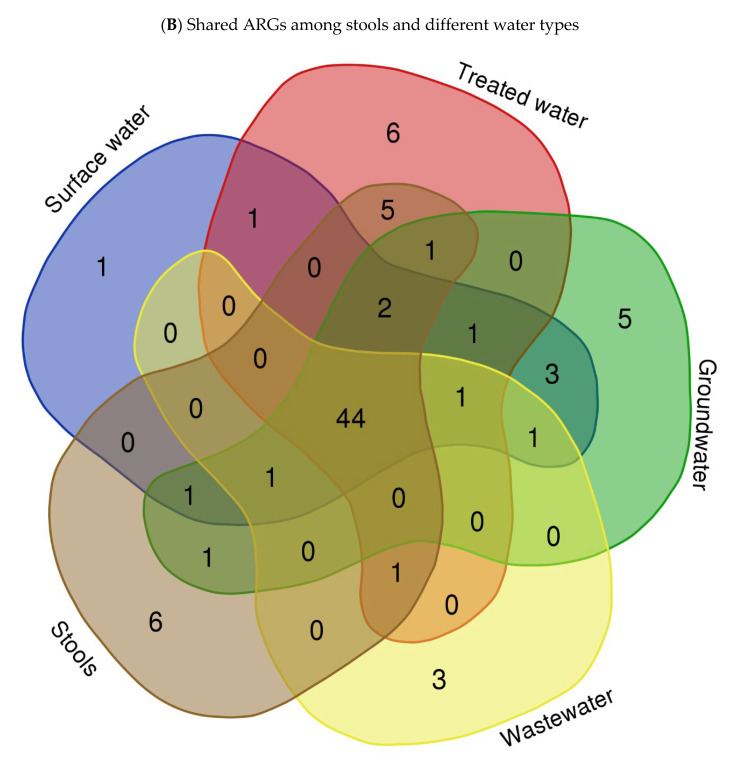
Heat map showing abundance of ARGs in different types of water (**A**) (with colour scale ranges from pale blue (low or zero abundance) to intermediate shades (moderate abundance) to dark/deep blue (high abundance))and Venn diagram showing shared ARGs among different stool samples and different types of water (**B**).

To explore the ARG content of each matrix, WGS sequences were queried against the Comprehensive Antibiotic Resistance Database (CARD) using BLASTx. The results of this analysis revealed a wide range of ARGs found in the different assessed matrices. We identified an average of 29, 178, 131, 29, 101, 188, 39, and 232 ARGs in isolates from springs (S), stool samples (SS), septic tank wastewater (SP), tap water at the point of use in the community (TWPC), rivers (R), household containers (HC), dams (D), and boreholes (B), respectively. Figure 3A shows the heat map of relative abundance of ARG profiles per assessed samples of the most abundant ARGs across all matrices sampled. In terms of ARG abundance, the highest abundance was observed for *mdtB* (*n* = 36), followed by *emrA* (*n* = 35), detected across all water sources. The *mdtC*, *emrK*, and *acrB* resistance genes were detected 32 times each in all matrices. The S, D, and TWPC samples were found to have the highest number of the *acrB* gene, followed by the *AcrS*, *baeR*, and *mdtP* genes. The dam (D) samples also showed a high number of *emrK*, *acrA*, and *evgA* genes. Among the identified ARGs, one of the most common ARGs (*acrA*) was detected in this study. The most frequent ARGs identified, like *mdtB*, *emrA*, and *acrB*, are principally related to multidrug efflux systems, which are implicated in resistance via the active exportation of antibiotics from the bacterial cell [5]. The core resistome was defined as shared ARGs found in all assessed matrices (Figure 3B). The core resistome was found to be 44 ARGs. Three (3) ARGs were shared between groundwater and surface water, while one (1) ARG was shared between surface water and drinking water at the household level and two (2) ARGs were shared among surface water, groundwater, wastewater, and household drinking water.

## 4. Discussion

The present study focused on the detection and identification of *E. coli* and *V. cholerae* strains. It compared the results of culturing, MALDI-TOF MS, and PCR-based detection of the main virulence-associated genes with WGS-derived findings of the virulome and resistome. These analyses were conducted in various types of water and stool samples. For pathogenic STEC, *E. coli* O157:H7, and *V. cholerae* isolates, culture-based methods revealed that the highest prevalence was found in stool samples with a prevalence rate of 32.6%, 30.4%, and 33.3%, respectively (Figure 1). This is to be expected, as *E. coli* is a ubiquitous human intestinal flora and is commonly excreted in stool, particularly with enteric infections. This finding is aligned with the fact that cholera is a diarrhoeal disease primarily transmitted through the faecal–oral route [28]. A study by Gwimbi et al. [29] also found a high prevalence of *E. coli* in samples from open wells and streams compared to unprotected springs. Surface water also showed a relatively high prevalence of presumptive pathogenic STEC (23.5%) and *V. cholerae* (22.8%). In Kenya, Sila [30] also reported that *V. cholerae* was mostly detected at high prevalence in Kauthulini River (36%) and Athi River (21%). These findings indicate that rivers may be contaminated with faecal matter, possibly from human or animal sources. Contaminated river water can pose a health risk to individuals who use the water source for drinking, bathing, or other purposes [31].

Springs, which are usually groundwater sources, can also be affected by contamination from nearby surface water or faulty sanitation systems. Groundwater and tap water at the point of use in the community had the lowest prevalence of presumptive pathogenic STEC, with rates of 3.8% and 2.7%, respectively. Similarly, for *Vibrio cholerae* isolates, groundwater had the lowest prevalence of isolates (1.4%), followed by tap water at the point of use in the community (2.3%) (Table 2). Boreholes are deep wells that extract water from underground water sources, and hence they typically have lower levels of contamination [32]. Similar results were obtained by Obanor et al. [33], where borehole water samples were found to have the lowest prevalence of *E. coli.* These findings are expected since these microbes are common inhabitants of the intestinal tracts of animals and can be shed in faeces, especially during episodes of gastrointestinal infections. It is in accordance with the fact that cholera is a diarrhoeal disease primarily transmitted through the faecal–oral route [28]. Similarly, Sila [30] also found that *V. cholerae* were mainly detected at high prevalence in Kenya, with 36% in Kauthulini River and 21% in Athi River. Consequently, the target bacterial isolates were subjected to MALDI-TOF MS Biotyper^®^ analysis. The high prevalence in stool samples suggested a significant level of faecal contamination.

The MALDI-TOF MS results revealed that more of the isolates were confirmed as STEC (36.8%, *n* = 204) than *E. coli* O157:H7 (15.7%, *n* = 87). For *V. cholerae*, 13.7% (*n* = 76) of the isolates were identified as *V. albensis*, a species closely related to *Vibrio cholerae*. These findings support the assumption that the presumptive *E. coli and V. cholerae* (identified as *V. albensis*) isolates in this study were indeed representative of pathogenic strains. *Vibrio albensis* is a non-O1 serovar *V. cholerae*, a luminescent bacterium that shares more than 70% of its DNA sequences with *V. cholerae* [34]. However, it is important to note that further characterisation and testing would be required to determine specific pathotypes within the STEC, *E. coli* O157:H7, and *V. cholerae* isolates. A small percentage of pathogenic STEC, *E. coli* O157:H7, and *V. cholerae* isolates were identified as other species: 1.4% (*n* = 5) as *Plesiomonas shigelloides*, 0.9% (*n* = 3) as *Proteus mirabilis*, *Aeromonas* spp. at 18.45% (*n* = 38), *Pseudomonas aeruginosa* at 9.71% (*n* = 20), *Pseudomonas mendocina* at 8.74 % (*n* = 18), *Morganella morganii* at 7.3% (*n* = 15), and *Enterobacter cloacae* at 5.8% (*n* = 12). These results suggest the presence of multiple bacterial species in water sources that may have pathogenic potential; for example, *Plesiomonas shigelloides* is known to cause diarrhoeal disease; *Proteus mirabilis* and *Morganella morganii* cause urinary tract infections; some species of *Aeromonas* cause gastrointestinal infections; and *Pseudomonas aeruginosa* cause urinary tract infections and respiratory tract infections [35]. Several isolates could not be identified; this is an indication of the presence of bacterial species that were not covered by the analysis or of potentially novel or unknown organisms. This is one of the general limitations of MALDI-TOF, as its database may not capture all the bacterial species, particularly emerging or rare ones [17]. Further investigation would be needed to determine the identity and significance of these unidentifiable isolates.

In this study, among the matrices tested, PCR results also showed that stool samples exhibited the highest prevalence of STEC, with 66% (*n* = 33) of samples testing positive (Table 5). In contrast, the highest prevalence of *Vibrio cholerae* being detected in hand-dug wells (Table S3 in Supplementary Data) indicates that hand-dug wells may serve as a reservoir for *V. cholerae*. Hand-dug wells are vulnerable to contamination, particularly if they are in areas with inadequate sanitation practices or in proximity to faecal sources [36]. The detection of *V. cholerae* in hand-dug wells raises concerns about the potential for cholera transmission if contaminated water from these wells is consumed without prior treatment. None of the tested springs showed the presence of virulence-associated genes of *Vibrio cholerae* (Table S3 in Supplementary Data). This finding suggested that the spring water tested in this study did not harbour pathogenic strains of *Vibrio cholerae*. Springs are typically considered a relatively safe source of drinking water, as groundwater is generally protected from surface contamination [37]. However, it is worth noting that the absence of virulence-associated genes does not completely rule out the presence of non-pathogenic or environmental strains of *Vibrio cholerae* in the springs. The presence of these virulence-associated genes indicates the need for proper sanitation and water treatment practices to prevent the transmission of waterborne diseases.

This study also found that a total of 34 isolates (9.1%) did not contain any of the target genes for either STEC, *E. coli* O157:H7, or *V. cholerae* when using conventional PCR. This suggested that these isolates were free from the specific pathogenic strains of these bacteria targeted in this study. Nevertheless, it is important to note that the absence of the target genes does not guarantee the absence of other potential pathogens or indicators of faecal contamination in those samples. Additionally, the absence of virulence-associated genes of *V. cholerae* in the tested spring water samples (Table S3 in Supplementary Data) is encouraging, indicating that these springs may pose a lower risk of *Vibrio cholerae* infection. However, it is crucial to regularly monitor and assess the quality of water sources to ensure public health and prevent waterborne illnesses.

The findings also showed the highest prevalence of the *stx1* gene in STEC was isolated from stored water [(*n* = 119 (72.1%)]; this gene is carried by STEC (Figure 1). This finding suggested a potential risk of contamination in household storage containers, which might be related to improper cleaning and maintenance practices, allowing for the survival and growth of pathogenic *E. coli* strains. The *fliCH_7_* gene encodes the H7 flagellar antigen, which is commonly linked to certain pathogenic *E. coli* strains [38]. The *hlyA* gene, associated with the production of haemolysin [39], exhibited the highest prevalence in STEC isolated from spring (S) samples [*n* = 10 (71.4%)]. This suggested that springs might be a source of STEC strains capable of producing haemolysin, which can cause damage to host cells and potentially contribute to the pathogenicity of *E. coli* infections. The *eae* gene, which is associated with the intimin protein involved in attaching and effacing lesions [40], exhibited the highest prevalence in STEC isolated from surface water [*n* = 8 (77.5%)]. The highest prevalence of the *fliCH_7_* gene was observed in *E. coli* O157:H7 isolated from stool samples [*n* = 45 (90%)]. The high prevalence of the *fliCH7* gene in stool samples may indicate the presence of pathogenic *E. coli* O157:H7 strains in the human intestinal tract and the potential for faecal–oral transmission. This finding demonstrates the potential presence of *E. coli* strains with attaching and effacing capabilities in hand-dug wells, which may pose a risk of infection if water is consumed without prior treatment.

Conversely, the *tcpA* gene carried by *V. cholerae* and the *eaeA* and *rfb_O157_* genes carried by *E. coli O157:H7* were the least frequently detected in the isolates from the various matrices (Figure 1). The low prevalence of these genes suggested a lower overall risk of infection with these specific pathogenic strains in the tested samples. Overall, these results provide insight into the distribution and prevalence of specific virulence-associated genes of pathogenic STEC, *E. coli* O157:H7, and *V. cholerae* strains in different matrices. Understanding the presence of these genes in various sources is valuable for assessing the potential health risks associated with water and environmental contamination and can aid in implementing appropriate control measures to mitigate these risks.

The average number of raw reads generated per sample was 1,635,181. It is worth noting that the genome coverage for all samples was reported to be greater than 30×, indicating that each position in the genome was covered by sequencing reads at least 30 times on average. Adequate genome coverage is crucial for the accurate assembly and analysis of genomic data, as it ensures a sufficient depth of sequencing to capture the genetic information present in the sample [41]. Hence, STEC emerged as the most frequently identified species in the water samples, also previously examined in the study area [42].

Whole-genome sequencing (WGS) and virulome analysis of *E. coli* isolated from the various types of water indicated variations in the virulence factor genes (VF genes) among the various water types. Virulome analysis identified a total of 153 VF genes across all the selected samples. The different sample types showed different numbers of VF genes. In Figure 3A, which shows the relative abundance of virulence factor genes in different water sources, the adhesion- and invasion-related genes, such as the *upaG* and the *ehaG* trimeric autotransporter adhesins (TAAs), were consistently the most frequently identified genes in most of the *E. coli* isolates, occurring 92 times. These TAAs have been characterised previously by Totsika et al. [43]. According to these authors, *ehaG* from enterohaemorrhagic *Escherichia coli* (EHEC) O157:H7 and *upaG* from uropathogenic *E. coli* (UPEC) were found to share similar properties for pathogenesis, but these TAAs also showed differences. While *ehaG* mediates binding to colorectal epithelial cells, *upaG* mediates binding to bladder epithelial cells. Moreover, the availability of genome sequencing through WGS has significantly increased the amount of information generated in laboratory testing using conventional methods. By sequencing the entire genome, researchers can uncover a broader range of virulence factors and genetic markers associated with diarrhoeal diseases [44]. In this case, the study identified specific VF genes, including *hcp1/tssD1*, *hlyE*, *mrkA*, and *mrkB*, which are known to be associated with diarrhoeal diseases.

The presence of these VF genes in various water sources provides additional evidence that these genes are widespread and have a significant impact on the pathogenicity of *E. coli.* The VF genes shared between stool samples and different water sources, as demonstrated in this study, have also been observed in other studies. A study by Ateba and Mbewe [45] explored the occurrence of virulence factor genes in *E. coli* isolates from various sources, including water and stools, and found a significant overlap between their virulence factor gene profiles. This implies a potential exchange of pathogenic strains and genes between animals and environmental reservoirs. It is important to note that the absence of VF gene sharing among surface water, groundwater, stools, and wastewater, as reported in this study, may suggest distinct sources and routes of contamination. These findings emphasise the importance of understanding the sources and transmission dynamics of pathogenic strains in water environments to mitigate the risks associated with waterborne diseases. However, human faecal contamination remains a major concern since it introduces enteric pathogens into water and increases the degree of exposure to infectious strains. These findings have implications for water quality management, public health protection, and the development of targeted interventions to reduce the risk of waterborne infections.

One of the objectives of this study was to explore the ARG content in each matrix and to identify the abundance and shared ARGs among them. The WGS analysis revealed a wide range of ARGs across isolates from different matrices (Figure 3). On average, this study identified 29, 178, 131, 29, 101, 188, 39, and 232 ARGs in the matrices S, SS, SP, TWPC, R, HC, D, and B, respectively. This reveals variations in the ARG content among the different water sources and sample types. The most abundant ARG was found to be *mdtB*, detected 36 times, followed by *emrA*, detected 35 times across all water sources. The prevalence of *mdtB* and *emrA* genes in these matrices implies that they are widely distributed and may play a significant role in antibiotic resistance within the bacterial populations present in the analysed water sources. These genes are associated with efflux pump systems, which are mechanisms that bacteria use to actively remove antibiotics from the cell, reducing their effectiveness [46].

It is important to record that the prevalence and significance of ARGs can vary depending on bacterial species, geographical locations, and other factors. This means that bacteria harbouring these ARGs can resist the effects of multiple classes of antibiotics, making it challenging to treat infections caused by these resistant strains. In this study, *acrA*, *acrD*, *acrE*, *acrS*, *baeR*, and *baeS* genes were widely distributed among the target *E. coli* strains in different matrices sampled and their presence is often associated with multidrug resistance. Understanding the prevalence and mechanisms of antibiotic resistance genes is crucial for the development of effective strategies to combat bacterial infections [47]. This knowledge can help in the selection of appropriate antibiotics and the development of new drugs or alternative treatment approaches to combat antibiotic-resistant bacteria.

This study also investigated the core resistome, which refers to the shared ARGs found in isolates from all assessed matrices. The core resistome was found to consist of 44 ARGs in isolates from various water matrices. Furthermore, the WGS analysis identified ARGs shared between different water sources. For example, three (3) ARGs were shared between isolates from groundwater and surface water (Figure 3B). Additionally, surface water, groundwater, wastewater, and household drinking water shared two ARGs (Figure 3B). These results provide insight into the diversity and abundance of ARGs in different water sources and highlight the presence of shared antibiotic resistance genes among these matrices. The findings are consistent with previous studies that have also reported variations in the presence of ARGs in different types of water. For instance, a study by Yang et al. [48] investigated ARGs in different water sources, from river water to tap water, and found differences in the abundance and composition of antibiotic resistance genes among the samples.

These data clearly demonstrate the value of WGS as a powerful tool that goes beyond traditional epidemiological methods, enabling a comprehensive and thorough detection of VF genes and ARGs of the studied pathogen such *E. coli*. In addition, in this study, it was shown that one ARG was shared between surface water and drinking water in households; the persistence of ARGs in HH drinking water highlights potential concerns regarding the spread of antibiotic resistance from one source to another (Figure 3B). It suggests that even if the water passes through treatment processes, which aim to remove harmful microorganisms, ARGs may remain present and pose a risk to public health. Understanding the ARG content and the distribution patterns of antibiotic resistance genes in water sources is crucial for addressing the issue of antibiotic resistance and developing strategies for water quality management and public health protection. It allows for targeted interventions to mitigate the spread of antibiotic resistance and reduce the potential risks associated with the presence of ARGs in the environment.

## 5. Conclusions

In conclusion, lack of or inadequate access to clean water and poor sanitation facilities in rural communities can lead to the occurrence of waterborne diseases. Pathogenic microorganisms are responsible for waterborne infections, which can be transferred from one water source to another if appropriate water treatment is not practised. This study’s findings highlight a high level of detection of Shiga toxin-producing *Escherichia coli*, *E. coli* O157:H7, and *Vibrio cholerae* in water and stool samples, emphasizing the potential health risks of water contamination. Although culture-based techniques and MALDI-TOF mass spectrometry are used for the initial identification, WGS provides more detailed information regarding the genetic makeup of the pathogens, such as the availability of antimicrobial resistance (AMR) genes and virulence factors (VFs). The existence of virulence genes such as *hcp1/tssD1*, *hlyE*, *mrkA*, and *mrkB* and antibiotic resistance determinants such as *acrA*, *acrD*, *acrE*, *acrS*, *baeR*, and *baeS* indicates the capacity for more aggressive disease and drug-related problems. The similarity of VF genes and ARGs across different matrices, i.e., stool and treated water samples, works to reinforce the potential survival of the pathogens and possible transmission through the water route. These findings support the need for ongoing surveillance, more effective water treatment methods, and strict public health practices to prevent waterborne disease outbreaks.

## Figures and Tables

**Table 1:** Primer sequences for the detection of STEC, *E. coli O157:H7*, and *V. cholera*.

Organism	Genes	Primer Sequence (F: Forward, R: Reverse) 5′ to 3′	Product Size (bp)	Annealing Temp (°C)	References
STEC	*stx1*	F-ATAAATCGCCATTCGTTGACTAC	180	55	[18]
		R-AGAACGCCCACTGAGATCATC			
	*stx2*	F-GGCACTGTCTGAAACTGCTCC	255	55	
		R-TCGCCAGTTATCTGACATTCTG			
	*Eae*	F-GACCCGGCACAAGCATAAGC	384	55	
		R-CCACCTGCAGCAACAAGAGG			
	*hlyA*	F-GCATCATCAAGCGTACGTTCC	534	55	
		R-AATGAGCCAAGCTGGTTAAGCT			
*E. coli O157:H7*	*rfb_O157_:H7*	F-CGGACATCCATGTGATATGG	259	56	[19]
		R-TTGCCTATGTACAGCTAATCC			
	*fliCH7*	F-TACCATCGCAAAAGCAACTCC	247	56	
		R-GTCGGCAACGTTAGTGATACC			
	*eaeA*	F-AAG CGA CTG AGG TCA CT	450	56	
		R-ACG CTG CTC ACT AGA TGT			
*V. cholerae*	*ctxA*	F-GGTCTTATGCCAGAGGACAG	219	50	[20]
		R-GTTGGGTGCAGTGGCTATAAC			
	*tcpA*	F-ATTCTTGGTGATCTCATGATAAGG	295	50	
		R-TTAATTCACCACAAATATCTGCC			
	*ompW*	F-CACCAAGAAGGTGACTTTATTGTG	588	50	
		R-GAACTTATAACCACCCGCG			
		R-ACCCAGTTTGCAGTTCCGAATGT			

**Table 2:** Culture-based detection of STEC, *E. coli* O157:H7, and *V. cholerae* in water and stool samples.

Matrices	Total Sample	Total Number of Samples Testing Positive (%)					
		STEC		*E. coli* O157:H7		*V. Cholerae*	
Surface water	136	32	(23.5%)	8	(5.9%)	31	(22.8%)
Groundwater	664	25	(3.8%)	20	(3.0%)	9	(1.4%)
Treated water *	640	17	(2.7%)	14	(2.2%)	15	(2.3%)
Stored water	1624	96	(5.9%)	30	(1.85%)	88	(5.4%)
Wastewater	104	9	(8.7%)	13	(12.5%)	18	(17.3%)
Stool	135	44	(32.6%)	41	(30.4%)	45	(33.3%)
Total	3303	223	(6.8%)	126	(3.8%)	206	6.2%)

Note: * treated and untreated water sample.

**Table 3:** Identification of *E. coli* and *V. cholerae* by MALDI-TOF in water and stool samples.

Matrices	Total Sample	Total Number of Samples Testing Positive (%)					
		STEC		*E. coli* O157:H7		*V. Cholerae*	
Surface water	71	10	(14.1%)	5	(0.7%)	7	(9.9%)
Groundwater	68	25	(36.8%)	19	(2.8%)	10	(14.7%)
Treated water	37	20	(54.1%)	6	(1.6%)	8	(21.6%)
Stored water *	209	89	(42.6%)	34	(1.6%)	23	(11.0%)
Wastewater	40	24	(60.0%)	9	(2.3%)	7	(17.5%)
Stools	130	36	(27.7%)	14	(1.1%)	21	(16.2%)
Total	555	204	(36.8%)	87	(1.6%)	76	(13.7%)

Note: * treated and untreated water sample.

**Table 4:** Summary statistics comparing the genome assemblies of the *Escherichia coli* population in various water types sampled.

Sample ID	Species	No. of Contigs	Total Length (bp)	No. of Sequence	N50	GC (%)	No. of Genes	GenBank Accession No.
B,MK1	*Escherichia coli*	1584	1,255,779	4243	801	51.81	3094	JASBBF000000000
B,MK2	*Escherichia coli*	1703	1,326,996	4829	774	52.55	3445	JASBBG000000000
B,MK3	*Escherichia coli*	2863	2,971,202	4815	1122	52.97	3838	JASBBH000000000
B,TM	*Escherichia coli*	1586	1,178,354	5527	738	50.71	3695	JASBBI000000000
B,TM1	*Escherichia coli*	1455	1,088,444	4993	741	52.11	3429	JASBBJ000000000
B,TM2	*Escherichia coli*	3507	3,968,282	5004	1224	52.07	4278	JASBBK000000000
B,TM3	*Escherichia coli*	2989	3,280,046	4779	1203	52.78	3925	JASBBL000000000
D,MK	*Escherichia coli*	3588	3,745,404	6211	1119	51.32	4860	JASBBM000000000
HC,CC4	*Escherichia coli*	1895	1,569,200	4783	852	52.71	3506	JASBBN000000000
HC,CC5	*Escherichia coli*	3385	3,542,931	6013	1134	52.11	4564	JASBBO000000000
HC,MK1	*Escherichia coli*	1663	1,241,949	5009	738	50.88	3557	JASBBP000000000
HC,MK3	*Escherichia coli*	1535	1,148,875	5144	741	52.18	3604	JASBBQ000000000
HC,MK	*Escherichia coli*	1588	1,195,056	5661	756	52.92	3824	JASBBR000000000
HC,TM4	*Escherichia coli*	1728	1,338,484	5087	774	52.48	3637	JASBBS000000000
R,CC1	*Escherichia coli*	2819	2,790,526	5045	1047	52.36	4022	JASBBT000000000
R,TM1	*Escherichia coli*	1638	1,175,496	6354	705	49.75	3996	JASBBU000000000
R,TM3	*Escherichia coli*	1611	1,280,091	4593	798	52.2	3270	JASBBV000000000
S,TM1	*Escherichia coli*	1883	1,512,031	5449	813	52.45	3573	JASBBW000000000
SP,CC1	*Escherichia coli*	1429	1,069,863	5260	753	51.81	3617	JASBBX000000000
SP,MK1	*Escherichia coli*	1787	1,476,801	4787	837	52.49	3370	JASBBY000000000
SP,MK2	*Escherichia coli*	3026	3,153,597	4333	1104	52.79	3205	JASBBZ000000000
SP,MK3	*Escherichia coli*	1624	1,247,824	4963	771	52.39	4016	JASBCA000000000
SS,CC1	*Escherichia coli*	2532	2,348,190	4842	966	52.71	3405	JASBCB000000000
SS,CC2	*Escherichia coli*	2454	2,372,235	4924	1011	52.3	3774	JASBCC000000000
SS,MK	*Escherichia coli*	3048	3,199,724	4544	1125	52.68	3462	JASBCD000000000
SS,MK2	*Escherichia coli*	1776	1,443,500	4962	831	52.32	3981	JASBCE000000000
SS,MK3	*Escherichia coli*	1461	1,060,329	4769	717	52.02	3493	JASBCF000000000
TWPC,CC1	*Escherichia coli*	1826	1,527,059	4486	846	53.03	3290	JASBCG000000000

Note: Makhado (MK), Thulamela (TM), Collins Chabane (CC), river (R), dam (D), spring (S), borehole (B), tap water at the point of use in the community (TWPC), household container (HC), septic tank wastewater (SP), and stool sample for humans and animals (SS).

**Table 5:** PCR detection of *E. coli* and *V. cholerae* virulence genes in water and stool samples.

Matrices	Total Sample	Total Number of Samples Testing Positive (%)					
		STEC		*E. coli* O157:H7		*V. Cholerae*	
Surface water	18	9	(50.0%)	5	(27.8%)	4	(22.2%)
Groundwater	66	23	(34.8%)	18	(27.3%)	8	(12.1%)
Treated water	35	19	(54.3%)	6	(17.1%)	6	(17.1%)
Stored water *	158	76	(48.1%)	43	(27.2%)	22	(13.9%)
Wastewater	40	20	(50.0%)	11	(27.5%)	7	(17.5%)
Stools	50	33	(66.0%)	12	(24.0%)	18	(36.0%)
Total	367	180	(49.0%)	95	(25.9%)	65	(17.7%)

Note: * treated and untreated water sample.

## Data Availability

The original contributions presented in this study are included in the article/Supplementary Materials. Further inquiries can be directed to the corresponding authors.

## Supplementary Materials

The following supporting information can be downloaded at: https://www.mdpi.com/article/10.3390/microorganisms13061373/s1, Table S1: Total number of samples collected during wet and dry season; Table S2: Water sample volume used for membrane filtration; Table S3: The prevalence of STEC, *E. coli* O157:H7 and *V. cholerae* isolates per matrix; Figure S1: Shiga-toxin *Escherichia coli* Gel 1 Note: lane M-gene ruler, 1-WS9, 2-WS41, 3-WS27, 4-WS27, 5-DS14, 6-SS1, 7-WS42, 8-DS7, 9-DS43, 10-WS6, 11-WS4, 12-DS5, 13-DS20, 14-DS51; Figure S2: *Escherichia coli* O157h:7 Gel 1. Note: lane M-gene ruler, 1-WO8, 2-DO7, 3-DO4, 4-D014, 5-DO15, 6-DO12, 7-DO1, 8-WO7, 9-WO6, 10-WO18, 11-WO23, 12-DO11, 13-DO2, 14-DO51; Figure S3: *Vibrio cholerae* Gel 1. Note: lane M-gene ruler, 1-VW43, 2-WV14, 3-VW12, 4-VW11, 5-WV16, 6-WV13, 7-WV52, 8-VW48, 9-WV91, 10-VW17, 11-WV54; Figure S4: *Vibrio cholerae* Gel 2. Note: lane M-gene ruler, 12-WV14, 13-WV42, 14-VW21, 15-WV34, 16-WV28, 17-WV40, 18-WV22, 19-WV4, 20-WV2, 21-WV30,22-WV23, 23-WV19, 24-VW8, 25-WV51, 26-WV39.
