# Supplementary material for: Identification, Comparison, and Profiling of Selected Diarrhoeagenic Pathogens from Diverse Water Sources and Human and Animal Faeces Using Whole-Genome Sequencing
Source: Microorganisms. 2025 Jun 12;13(6):1373. doi: 10.3390/microorganisms13061373 (PMC12196432; doi:10.3390/microorganisms13061373)
Supplement: Supplementary file 1 [file microorganisms-13-01373-s001.zip › microorganisms-3654511-supplementary.pdf]

Supplementary Information for Murei and Momba

Arinao **Murei**[1] • Maggy Ndombo Benteke **Momba**[1]*

[1] Department of Environmental, Water and Earth Sciences, Tshwane University of Technology, Arcadia Campus, Private BagX680, Pretoria 0001, South Africa.

Table S1: Total number of samples collected during wet and dry season

	**Sampling point**	**Thulamela LM**		**Collins-Chabane LM**		**Makhado LM**		**Tota l**
Number of villages		6		4		4		
		Wet	Dry	Wet	Dry	Wet	Dry	
	R	48	48	8	8	0*	0*	112
	D	8	8	0*	0*	4	4	24
Catchment	S	20	20	0*	0*	8	8	56
	B	112	112	4	4	180	180	592
	DUG	8	8	0*	0*	0*	0*	16
Household	TWPC	102	95	182	178	44	39	640
	HC	376	376	308	300	132	132	1624
Septic tank	SP	16	16	0*	0*	36	36	104
Stools	SS	24	19	6	6	40	40	135
Total								**3303**

Note: * Not detected. Where LM (local municipality), R (river), D (dam), S (springs), B (boreholes), DUG (dug wells), TWPC (tap water at the point of use in the community), HC (household container), SP (septic tank wastewater), and SS (stool samples of humans and animals)

Table S2: Water sample volume used for membrane filtration.

**Water source**	**Volume (X) to be filtered mL**				
	100	50	10	1	0.1
Rivers				X	X

Dams			X	X
Springs	X	X		
Boreholes	X			
Hand-dug wells		X		

Table S3: The prevalence of STEC, *E. coli* O157:H7 and *V. cholerae* isolates per matrix.

Matrices	Total number of samples tested	Total number of samples testing positive (%)					
		**STEC**		***E. coli* o157**		***V. Cholerae***	
R	112	30	(26.8%)	6	(5.4%)	30	(26.8%)
D	24	2	(8.3%)	2	(8.3%)	1	(4.2%)
B	592	22	(3.7%)	10	(1.7%)	1	(0.2%)
S	56	8	(14.3%)	0	(0.0%)	0	(0%)
DUG	16	3	(18.8%)	2	(12.5%)	8	(50%)
TWPC	640	17	(2.7%)	14	(2.2%)	15	(2.3%)
HC	1624	96	(5.9%)	30	(1.8%)	88	(5.4%)
SS	104	9	(8.7%)	13	(12.5%)	18	(17.3%)
SP	135	44	(32.6%)	41	(30.4%)	45	(33.3%)

Detection of *E. coli*

180 pb represents *stx1* gene

255 bp represents *stx2* gene

384 bp represents *eae* gene

534 bp represents *hlyA* gene

259 bp represents *rfbO157* genes

247 bp represents *FlicH7* genes

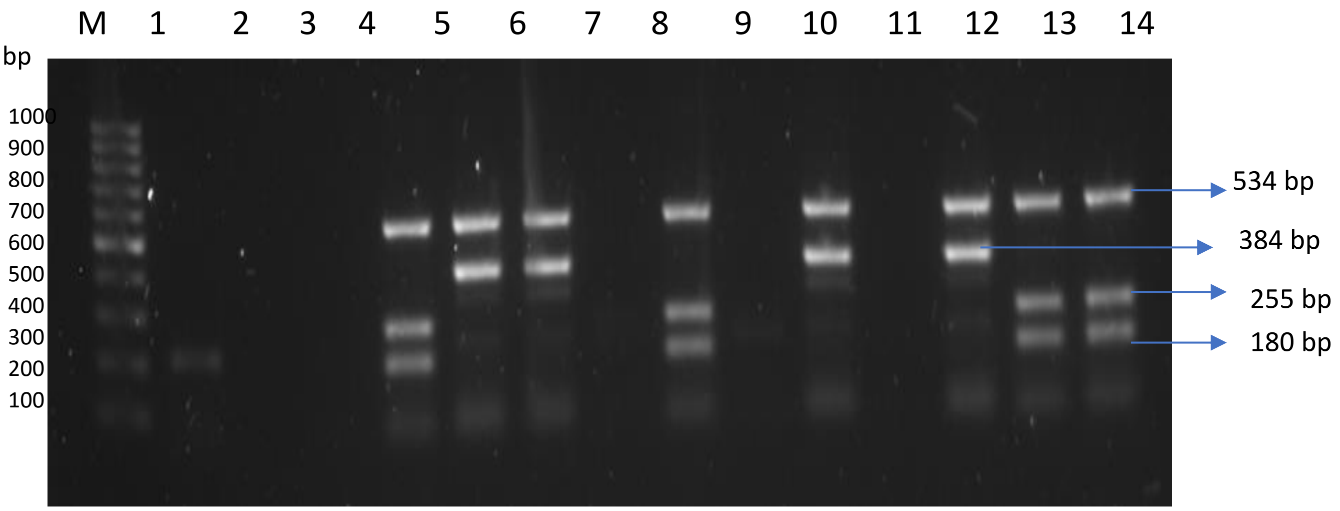

Figure S1: Shiga-toxin *Escherichia coli* Gel 1 Note: lane M-gene ruler, 1-WS9, 2-WS41, 3-WS27, 4-WS27, 5-DS14, 6-SS1, 7-WS42, 8-DS7, 9-DS43, 10-WS6, 11-WS4, 12-DS5, 13-DS20, 14-DS51

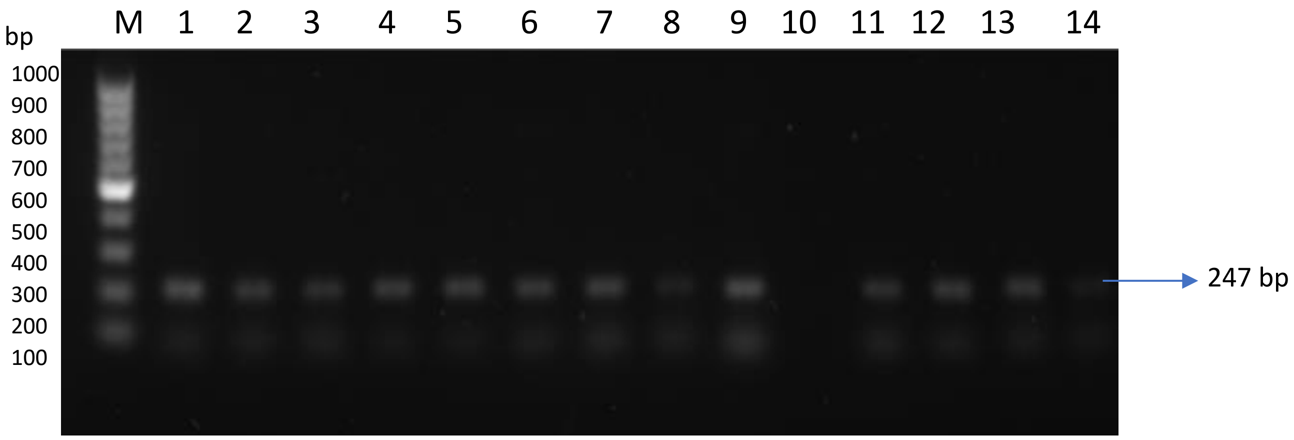

Figure S2: *Escherichia coli* O157h:7 Gel 1. Note: lane M-gene ruler, 1-WO8, 2-DO7, 3-DO4, 4-D014, 5-DO15, 6-DO12, 7-DO1, 8-WO7, 9-WO6, 10-WO18, 11-WO23, 12-DO11, 13-DO2, 14-DO51

Detection of *V. cholerae*

588 bp represents the *OmpW* genes

219 bp represents the *ctxA* genes

295 bp represents *tcpA* genes

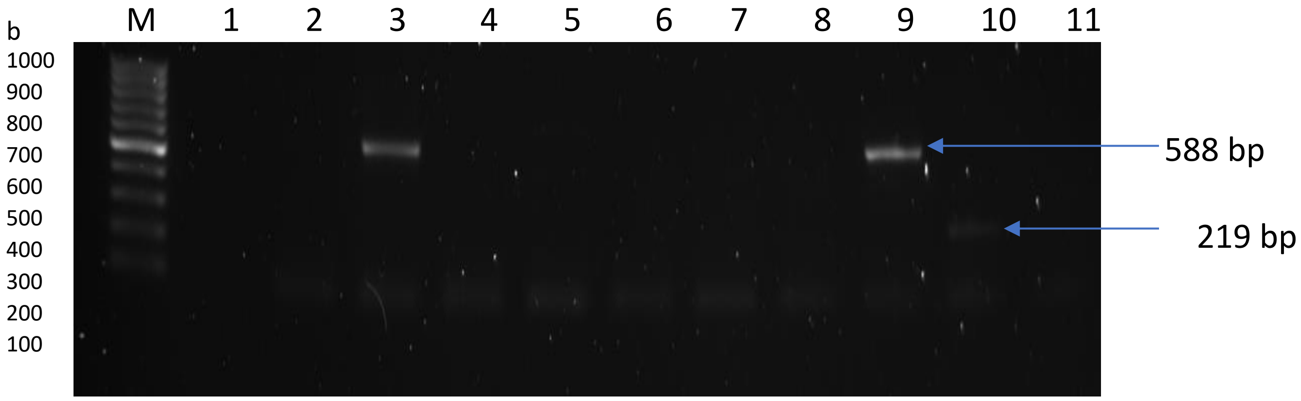

Figure S3*: Vibrio cholerae* Gel 1. Note: lane M-gene ruler, 1-VW43, 2-WV14, 3-VW12, 4-VW11, 5-WV16, 6-WV13, 7-WV52, 8-VW48, 9-WV91, 10-VW17, 11-WV54

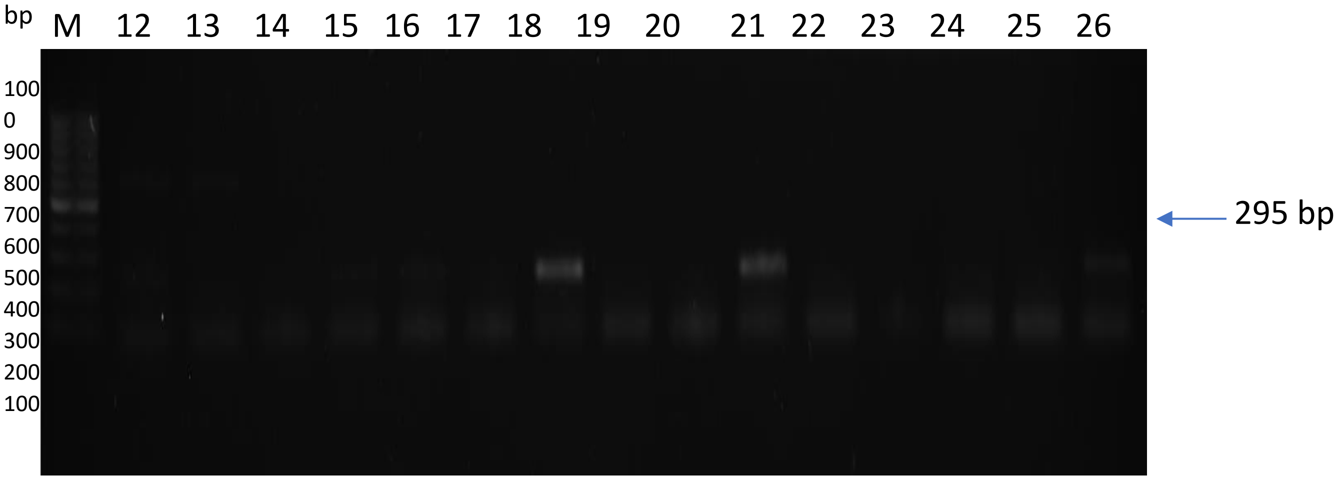

Figure S4*: Vibrio cholerae* Gel 2. Note: lane M-gene ruler, 12-WV14, 13- WV42, 14-VW21, 15-WV34, 16- WV28, 17- WV40, 18- WV22, 19- WV4, 20- WV2, 21- WV30,22- WV23, 23- WV19, 24-
